# Genetic barcoding reveals clonal dominance in iPSC-derived mesenchymal stromal cells

**DOI:** 10.1186/s13287-020-01619-5

**Published:** 2020-03-05

**Authors:** Jonathan Hollmann, Johanna Brecht, Roman Goetzke, Julia Franzen, Anton Selich, Marco Schmidt, Monika Eipel, Alina Ostrowska, Jan Hapala, Eduardo Fernandez-Rebollo, Gerhard Müller-Newen, Michael Rothe, Thomas Eggermann, Martin Zenke, Wolfgang Wagner

**Affiliations:** 1 Helmholtz Institute for Biomedical Engineering, Stem Cell Biology and Cellular Engineering, RWTH Aachen University Medical School, Pauwelsstrasse 20, 52074 Aachen, Germany; 2 Institute of Experimental Hematology, Hannover Medical School, 30625 Hannover, Germany; 3 Institute of Biochemistry and Molecular Biology, RWTH Aachen University, 52074 Aachen, Germany; 4 Institute of Human Genetics, RWTH Aachen University Hospital, 52074 Aachen, Germany; 5 Institute for Biomedical Engineering – Cell Biology, RWTH Aachen University Medical School, Aachen, Germany

**Keywords:** Mesenchymal stromal cells, Induced pluripotent stem cells, Clonality, RGB marking, Genetic barcoding, Limiting dilution, Epigenetic, DNA methylation

## Abstract

**Background:**

The use of mesenchymal stromal cells (MSCs) for research and clinical application is hampered by cellular heterogeneity and replicative senescence. Generation of MSC-like cells from induced pluripotent stem cells (iPSCs) may circumvent these limitations, and such iPSC-derived MSCs (iMSCs) are already tested in clinical trials. So far, a comparison of MSCs and iMSCs was particularly addressed in bulk culture. Despite the high hopes in cellular therapy, only little is known how the composition of different subclones changes in these cell preparations during culture expansion.

**Methods:**

In this study, we used multicolor lentiviral genetic barcoding for the marking of individual cells within cell preparations. Based on this, we could track the clonal composition of syngenic MSCs, iPSCs, and iMSCs during culture expansion. Furthermore, we analyzed DNA methylation patterns at senescence-associated genomic regions by barcoded bisulfite amplicon sequencing. The proliferation and differentiation capacities of individual subclones within MSCs and iMSCs were investigated with limiting dilution assays.

**Results:**

Overall, the clonal composition of primary MSCs and iPSCs gradually declined during expansion. In contrast, iMSCs became oligoclonal early during differentiation, indicating that they were derived from few individual iPSCs. This dominant clonal outgrowth of iMSCs was not associated with changes in chromosomal copy number variation. Furthermore, clonal dynamics were not clearly reflected by stochastically acquired DNA methylation patterns. Limiting dilution assays revealed that iMSCs are heterogeneous in colony formation and in vitro differentiation potential, while this was even more pronounced in primary MSCs.

**Conclusions:**

Our results indicate that the subclonal diversity of MSCs and iPSCs declines gradually during in vitro culture, whereas derivation of iMSCs may stem from few individual iPSCs. Differentiation regimen needs to be further optimized to achieve homogeneous differentiation of iPSCs towards iMSCs.

## Background

Mesenchymal stromal cells (MSCs) comprise various subpopulations, which are reflected by differences in proliferation, morphology, growth pattern, immunophenotype, and differentiation potential [1]. Only a subset of multipotent MSCs is capable of in vitro differentiation towards osteogenic, chondrogenic, and adipogenic lineages [1–3]. Furthermore, only a few cells possess the proliferative potential to form fibroblastic colony-forming units (CFU-f) [1, 2, 4, 5]. Little is known about how the clonal composition changes during culture expansion, albeit this is of considerable relevance with regard to the high hopes for MSCs in a multitude of clinical trials.

Multicolor lentiviral genetic barcode labeling (RGB-BC) enables the marking of individual cells to track their progeny during culture expansion [6]. The lentiviral vectors can code for different fluorescent proteins—e.g., red, green, and blue (RGB)—to visualize clonal cell expansion in vitro and in vivo. However, the resolution in the detection of individual subclones with three different RGB vectors is limited to about eight different color combinations. This resolution can be massively increased by introducing complex genetic barcodes into the lentiviral constructs. These barcodes provide unique molecular identifiers (UMI) for subsequent deep sequencing analysis to track the progeny of individual clones. If labeling with RGB and UMI is combined, the barcodes can comprise additional base pairs to reflect the color of the corresponding lentiviral construct [6]. This cell marking technique has been used before to follow the clonal dynamics during culture isolation of MSCs upon marking in umbilical cord pieces [7, 8]. A remarkable reduction in clonal complexity occurred already during the first three passages, when non-MSC cells disappeared from the culture [7]. Given the heterogeneity of MSCs, it may be anticipated that the clonal diversity within established in vitro cell preparations, e.g. from bone marrow, might also decline during culture expansion.

An alternative approach to isolation of primary MSCs is the derivation of MSC-like cells from induced pluripotent stem cells (iPSCs), referred to as iPSC-derived MSCs (iMSCs) [9–11]. In their pluripotent state, iPSCs are expanded to high cell numbers without any signs of replicative senescence—thus they provide an essentially unlimited source for the generation of high numbers of iMSCs. It may be anticipated that the production of iMSCs can be better standardized and provide more homogeneous cell preparations than primary MSCs. On the other hand, differentiation of iPSCs towards iMSCs does not necessarily occur simultaneously, and it remains to be demonstrated if iMSCs are indeed functionally more homogeneous. So far, comparison of MSCs and iMSCs primarily focused on the similarities in cell surface marker expression, cellular morphology, differentiation potential, and immunomodulatory function—and these aspects still deserve further elucidation. In fact, the first clinical trials with iMSCs are already ongoing for steroid-refractory graft versus host disease [12, 13]. With regard to the high demands of cell safety in therapeutic application, it is therefore crucial to better understand the clonal dynamics and the heterogeneity within iMSCs.

Long-term culture of MSCs can be tracked by epigenetic modifications. Various genomic regions become continuously hyper- or hypomethylated during culture expansion [14, 15]. We have previously shown that these modifications are independently acquired at neighboring CG dinucleotides (CpGs) [16, 17]. A predominant DNA methylation pattern may, therefore, reveal the prevalence of dominant subclones [18].

In this study, we monitored changes in the clonal composition during culture expansion of primary MSCs, iPSCs, and iMSCs with genetic barcoding and analysis of DNA methylation patterns. Furthermore, we compared the functional heterogeneity within MSC and iMSC preparations by analyzing subclonal proliferation and differentiation capacities in limiting dilution assays.

## Methods

### Cell culture

Primary MSCs were isolated from femoral bone marrow of three different patients after orthopedic surgery [19]. Reprogramming of MSCs (at passage 1) into iPSCs was performed with episomal plasmids [20]. Pluripotency of single-colony-derived iPSCs was validated by in vitro differentiation and Epi-Pluri-Score analysis (Cygenia GmbH, Aachen, Germany) [21]. For re-differentiation of iPSCs towards iMSCs, we adopted the protocol of Frobel et al. [9], which is based on the same culture medium that was used in the initial isolation of MSCs.

Culture medium for MSCs consisted of Dulbecco’s modified Eagle’s medium (DMEM; 1 g/L glucose; PAA, Pasching, Austria) with 1% penicillin/streptomycin (10,000 U/mL; Thermo Fisher Scientific, Waltham, USA), 1% L-glutamine (200 mM; Thermo Fisher Scientific), 0.1% heparin (5000 lU/mL; Ratiopharm, Ulm, Germany), and 10% pooled human platelet lysate [22]. The medium was changed every 3 days and cells were passaged at subconfluent growth by trypsinization (Trypsin/EDTA 0.25%; Thermo Fisher Scientific) and splitted 1:3 to 1:6.

iPSCs were cultured on Vitronectin XF-coated (Stem Cell Technologies, Vancouver, Canada) 6-well plates in StemMACS iPS-Brew XF (Miltenyi Biotec, Bergisch Gladbach, Germany). The medium change was performed every day and cells were passaged at 70% confluency every 3 to 5 days.

iMSCs were cultured in the culture medium for MSCs on 0.1% gelatin-coated (Merck, Darmstadt, Germany) 6-well plates. Medium changes and passaging were performed as described for primary MSCs at seeding densities of 10,000 cells/cm^2^.

### Immunophenotypic analysis

Immunophenotype of MSCs and iMSCs was analyzed with a fluorescence-activated cell sorter (FACS) Canto II (BD Biosciences, Franklin Lakes, USA) after staining with the following antibodies, as described before [23]: CD29 phycoerythrin (PE; clone MAR4; BD), CD34 allophycocyanin (APC; clone 581; BD), CD31 PE (clone WM59; BD), CD45 APC (clone HI30; BD), CD105 fluorescein isothiocyanate (FITC; clone MEM-226; ImmunoTools, Friesoythe, Germany), CD90 APC (clone 5E10; BD), CD73 PE (clone AD2; BD), and CD14 APC (clone M5E2; BD).

### Analysis of CFU-f

Primary MSCs and iMSCs were trypsinized and seeded in limiting dilutions at 30, 10, 3, and 1 cells/well in 4 × 96-well plates (96 replicas/dilution step). Cells were maintained under standard MSC culture conditions without passaging and wells were scored for 50% confluent growth after 15 days. Based on Poisson statistics, CFU-f frequencies were calculated using the L-Calc Limiting Dilution Software (Stem Cell Technologies, Vancouver, Canada) and probabilities for monoclonality were estimated as described before [1]. Subsequently, the multi-well plates were differentiated towards adipogenic or osteogenic lineage.

### In vitro differentiation of MSCs and iMSCs

Adipogenic, osteogenic, and chondrogenic differentiation was induced in MSCs and iMSCs as described in our previous work [1, 24]. Chondrogenic differentiation was induced in cell pellets, whereas adipogenic and osteogenic differentiation were induced in monolayers. For the analysis of differentiation in the limiting dilutions (96-well plates), we only considered wells at dilutions that were probably single-cell derived (probability > 50%) [1].

After 14 days of adipogenic differentiation, cells were stained for fat droplets with BODIPY (4,4-difluoro-1,2,5,7,8-pentamethyl-4-bora-3a,4a-diaza-s-indacene; Invitrogen, Carlsbad, USA) and counterstained for nuclei with Hoechst. Fluorescence microscopy pictures were acquired for representative areas of each well. Percentages of differentiated cells were estimated by counting nuclei and fat droplet containing cells with ImageJ, FIJI cookbook nucleus counter plugin, and FIJI Cell Counter plugin (https://imagej.net/Fiji; https://imagej.net/Cookbook) [22, 25]. However, because of the differences in lipid vesicle size of MSCs and iMSCs and therefore different exposure times in fluorescence microscopy, a direct comparison was hampered—thus, we did not statistically compare these results.

After 18 days of osteogenic differentiation, the cells were stained with Alizarin Red S (Sigma-Aldrich, St. Louis, USA) for calcium precipitates and semi-quantified with a Tecan Infinite 200 plate-reader (Tecan Group AG, Switzerland) at λ405 nm. For comparison in our limiting dilution experiments, wells with background normalized absorption values > 0.4 were considered positive for calcium phosphate precipitates.

### Generation of barcoded RGB vector library and virus production

LeGO vectors (plasmids pRRL-PPT-CBX3-EFS-Cerulean-P2A-Puro, pRRL-PPT-CBX3-EFS-mCherry-P2A-Puro, pRRL-PPT-CBX3-EFS-Venus-P2A-Puro) of Selich et al. [8], expressing the three fluorescent proteins mCherry (red), Venus (yellow-green), and Cerulean (blue), were equipped with an additional puromycin resistance gene to allow the selection of transduced cells. The barcodes contained color-specific nucleotide sequences, as well as random nucleotides specific for complex genetic barcodes (Additional file 1; Additional file 2) [6, 7]. Virus particles, pseudotyped with vesicular stomatitis virus G glycoprotein (VSV-G; plasmid pMD2.G; Addgene, Watertown, USA), were produced using HEK293T cells as packaging cells and a second-generation lentivirus packaging system (plasmid psPAX2; Addgene) [26, 27]. Virus-containing supernatants were kept frozen at − 80 °C until further usage.

### Transduction and puromycin selection

Transduction of primary MSCs was performed at passage 2 (50% confluent growth) on 6-well plates. Standard culture medium was then replaced by 293T Cell Medium supplemented with equal amounts of virus supernatants and 8 μg/mL polybrene (Sigma-Aldrich, St. Louis, USA) for 24 h [28]. Puromycin selection was performed for 3 to 4 days by the addition of increasing doses of puromycin (1 or 1.6 μg/mL).

For transduction of iPSCs, the cells were passaged to Matrigel-coated (Corning, NY, USA) 6-well plates at a ratio of 1:3 after reaching 70–80% confluency. The standard iPSC culture medium was supplemented with equal amounts of virus supernatants and 8 μg/mL polybrene for 3 h. After 24 h of cell culture, virus supernatants and polybrene were added for another 3 h. Puromycin selection was performed 48 h after transduction by the addition of increasing doses of puromycin (0.4–0.8 μg/mL) for 4 days.

### Flowcytometric analysis of RGB labels and fluorescence microscopy

Cell samples for flow cytometry were collected during passaging once per week. MSCs and iMSCs were detached by trypsin (Trypsin/EDTA 0.25%; Thermo Fisher Scientific, Waltham, USA), whereas iPSCs were harvested with Accutase (Stem Cell Technologies, Vancouver, Canada). Fixation of cells was performed with 2% paraformaldehyde (PFA; Carl Roth, Karlsruhe, Germany) for 15 min at room temperature. After two washing steps in phosphate-buffered saline (PBS; Thermo Fisher Scientific, Waltham, USA), cells were kept at 4 °C and analyzed using a BD LSR Fortessa (BD Biosciences, Franklin Lakes, USA) with the same settings for all cell types and time points. The following lasers and filters were used for Cerulean, Venus, and mCherry, respectively: 405 nm laser and 525/50 filters, 488 nm laser and 530/30 filters, and 561 nm laser and 575/26 filters. Data was analyzed with FlowJo software 10.4.2 (FlowJo, LLC, Ashland, USA). We used consistent gates for forward- and side-scatter over subsequent passages. To estimate fluorescence thresholds, we utilized iPSCs, which were transfected with individual fluorochromes. However, particularly at low-signal intensity, the fluorescence of other fluorochromes cannot be excluded. The fraction of non-fluorescent cells may therefore either be attributed to the relatively conservative thresholds or to gene silencing. Fluorescence images were acquired with an LSM 710 confocal microscope (Carl Zeiss, Oberkochen, Germany).

### DNA isolation and sequencing of barcodes

DNA was isolated with the NucleoSpin Tissue kit (Macherey-Nagel, Düren, Germany). For next-generation sequencing, barcodes were amplified and labeled with handle-sequences by a first PCR step with the primers Handle-BC-PCR-FW and Handle-BC-PCR-RV (Additional file 3) using the PyroMark PCR Kit (Qiagen, Hilden, Germany). PCR products were pooled and purified with Agencourt AMPure XP beads (Beckman Coulter, Brea, USA). In a second PCR step, Illumina sample barcodes were added to each sample using one common and respective barcode primers listed in Additional file 3 (adapted from NEXTflexTM 16S V1-V3 Amplicon Seq Kit, Bioo Scientific, Austin, USA). After quantification with a Qubit 2.0 (ThermoFisher Scientific, Waltham, USA) and equimolar pooling of the samples, final purification of PCR products > 200 bp was performed with the Select-A-Size DNA Clean & Concentrator kit (Zymo Research, Irvine, USA). Library preparation for sequencing was performed following the MiSeq System Denature and Dilute Libraries Guide to produce a 10-pM library, with a 15% spike in of PhiX Control and the MiSeq Reagent Kit V2 (all from Illumina, San Diego, USA). Libraries were sequenced in 250 bp paired-end mode on a MiSeq Illumina Sequencer.

### Processing of sequencing data

Processing of RGB-BC sequencing results was performed with custom scripts. First, cutadapt [29] (parameters -g ACCATCTAGA...CTCGAGACTG -G CAGTCTCGAG...TCTAGATGGT -e 0.2) was used to remove the read ends delimited by the primer sequences ACCATCTAGA and CTCGAGACTG. For each read, the color used was identified based on the barcode sequence, removing reads without barcode. The area graphs were produced with the ggplot2 [30] library in R. For each barcode the number of its occurrence (number of reads containing the barcode) per individual, cell type, and time point was plotted as the percentage of the total barcode reads. The Shannon-Index was calculated with the R package vegan [31].

### Barcoded bisulfite amplicon sequencing

The analysis of DNA methylation patterns in the amplicons of *GRM7*, *CASR*, and *PDE4C* was performed by bisulfite barcoded amplicon sequencing and analyzed as described before [16, 18].

### Copy number variation (CNV) analysis

Isolated DNA from iPSCs and corresponding iMSCs of all three donors were analyzed by single nucleotide polymorphism (SNP) array typing (CytoScan HD Array; Affymetrix, CA, USA) for CNV detection. All CNVs were considered that reached a threshold of > 200 kb with a mean marker distance of < 5 kb in at least one sample of either iPSCs or iMSCs.

### Data processing and statistics

Results of the limiting dilution assays are presented as single data points and grand mean and diagrams were created with GraphPad Prism 8 (GraphPad Software Inc., San Diego, USA). “*N*” represents the number of independent biological replicas and “*n*” the number of technical replicas. Statistical significance (*P*) was estimated with the two-tailed, unpaired Student’s *t* test.

## Results

### Genetic barcoding reveals gradual decline in clonal diversity during the expansion of primary MSCs

**Fig. 1:**
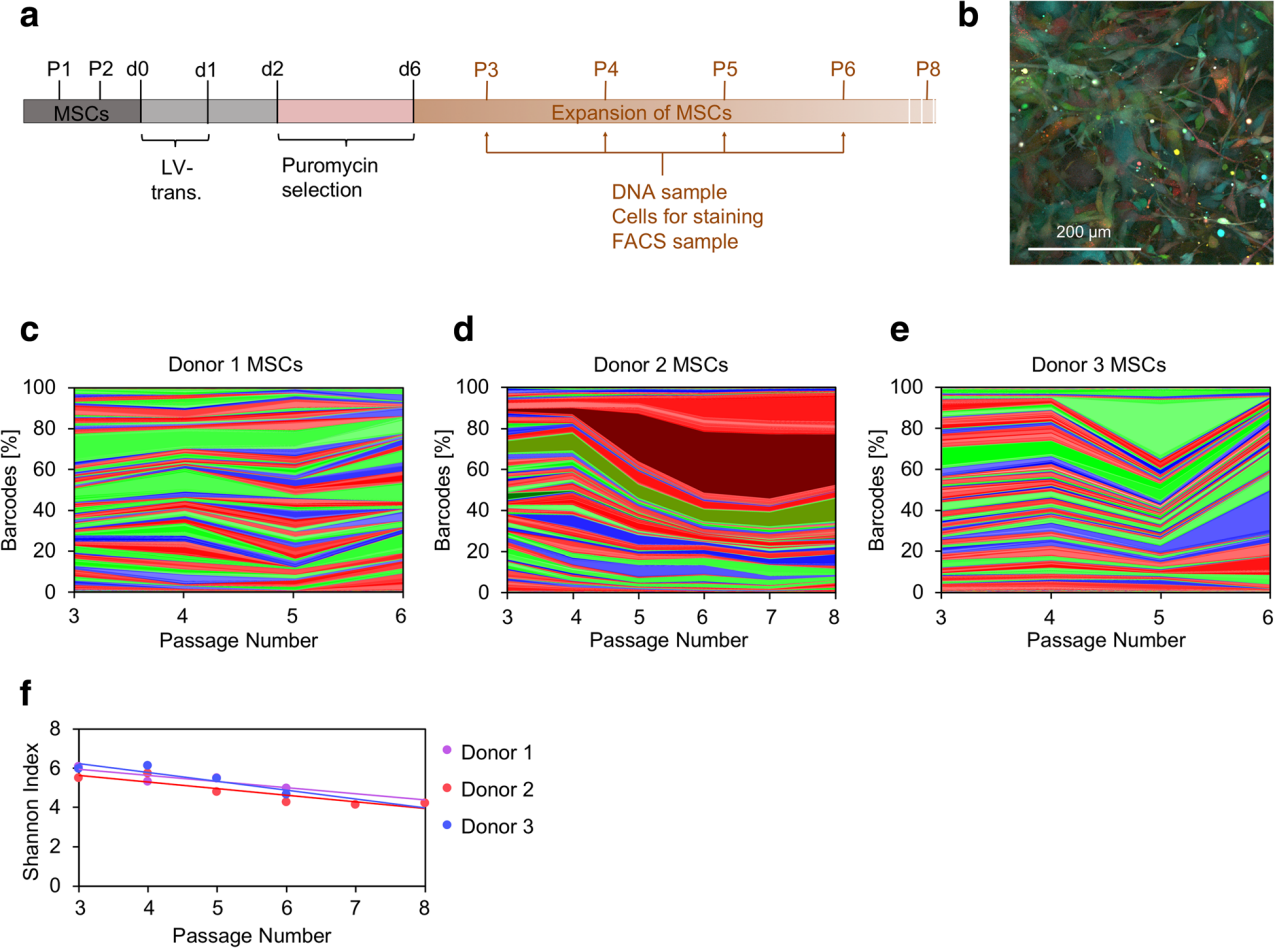
Clonal complexity declines during the culture of primary MSCs. **a** Schematic presentation of the experimental workflow. Lentiviral transduction (LV-trans.) with RGB-BC LeGO vectors, puromycin selection, culture expansion, and sampling of primary MSCs. P, passage; d, day. **b** Exemplary fluorescence microscopy image of RGB-marked MSCs. **c**–**e** Sequencing results of barcodes (colors indicate the corresponding fluorochrome of the vector construct). The contribution of each specific barcode is represented as a percentage of the total number of barcode reads. Changes were analyzed at consecutive passages for each donor. **f** Shannon Index indicates decline of subclone-diversity during culture expansion of MSCs

To track the clonal composition of primary MSCs over subsequent passages, we used lentiviral RGB-marking with genetic barcoding (Additional file 2a). Bone marrow-derived MSCs of three donors were transduced at passage 2 (P2) after isolation and selected for puromycin resistance (Fig. 1a). Transduced cells expressed a combination of the three fluorescent proteins mCherry (red), Venus (yellow-green), and Cerulean (blue) (Fig. 1b). Flow cytometry indicated that the color composition remained relatively stable over four to six consecutive passages (36–66 days) until cells stopped proliferating (Additional file 2b-d). Subsequently, we analyzed the genetic barcodes by deep sequencing and the results for the fluorophore-specific barcodes were consistent with flow cytometry. The proportions of subclones remained relatively stable throughout culture, without apparent clonal dominance, apart from some barcodes with mCherry label that accumulated in donor 2 (Fig. 1c–e). The Shannon Index, describing biological diversity, was high and decreased only moderately for all three donors (Fig. 1f), which indicates that the clonal heterogeneity gradually declined during the culture expansion of primary MSCs.

### The clonal composition of iPSCs remains relatively stable during expansion

**Fig. 2:**
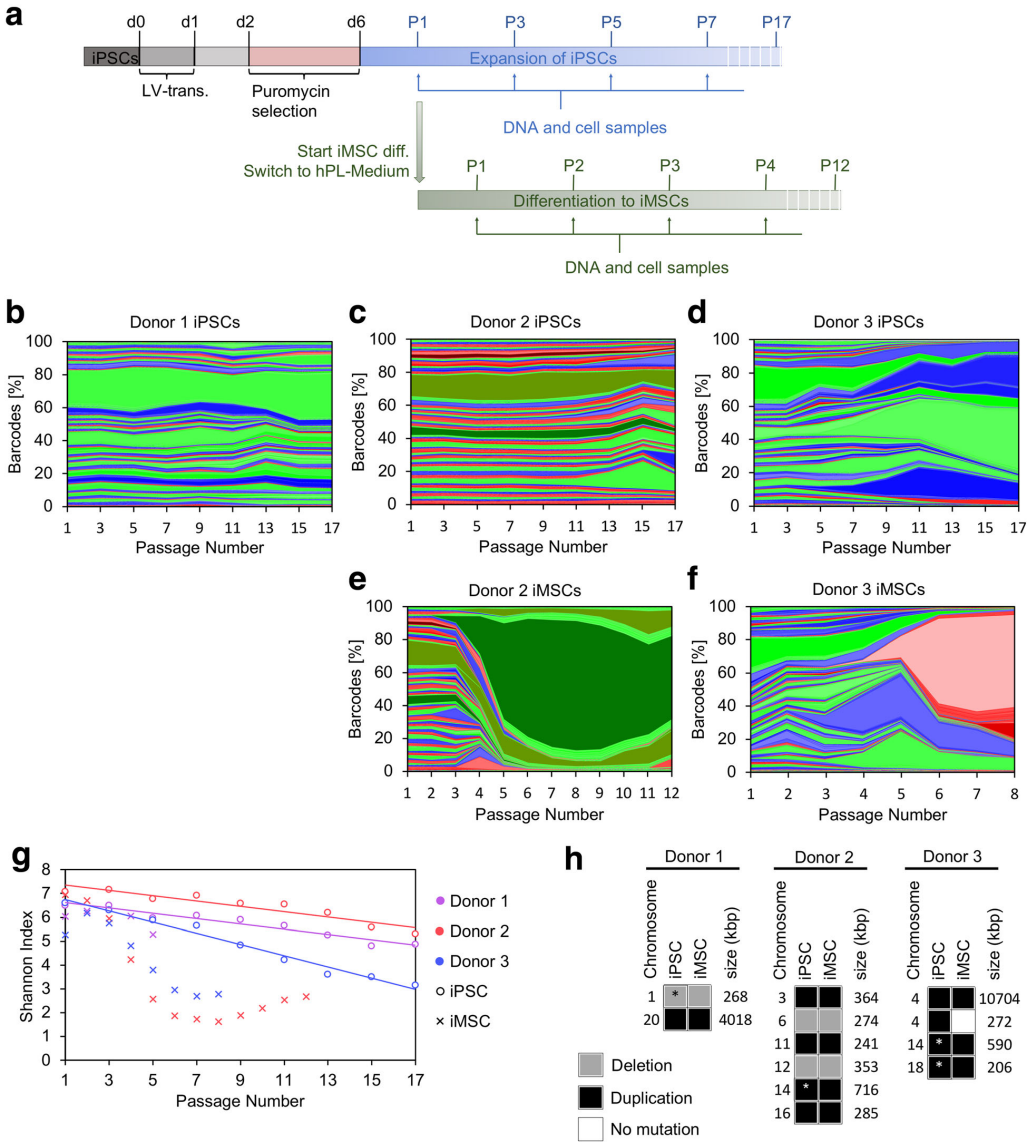
Generation of iMSCs is based on few dominant subclones. **a** Schematic presentation of the experimental workflow with lentiviral transduction (LV-trans.) of iPSCs. After puromycin selection, the genetic labelling with RGB-BC was either analyzed during long-term culture of iPSCs, or upon differentiation towards iMSCs. **b**–**d** Sequencing results of barcodes reveal a moderate decline in the composition of different barcodes during the expansion of iPSCs (colors are indicative for the corresponding fluorochrome of each barcode). The contribution of each specific barcode is represented as a percentage of the total number of barcode reads. Changes were analyzed at consecutive passages for each donor. **e**–**f** In analogy, the composition of barcodes was tracked during differentiation towards and expansion of iMSCs (only successful for iPSCs of donors 2 and 3). Both donors revealed dominant subclones that emerged during generation of iMSCs. **g** Shannon Index measuring subclone-diversity. **h** CNVs in iPSCs and iMSCs of all three donors. SNP array analysis was performed with DNA samples of iPSCs (P1) and iMSCs (donor 1, P2; donor 2, P11; donor 3, P6). Chromosomal location, and the largest size of the variants (kbp) in either iPSCs or iMSCs are indicated for gains (black) and losses (gray) of more than 200 kbp. *Chromosomal locations where the corresponding mutation was present, but below the threshold, see also Additional file 6`

Despite their uniform appearance, there is also marked heterogeneity within colonies of iPSCs [32, 33]. Therefore, we analyzed if specific subclones become dominant during serial passaging of iPSCs. The three MSC preparations were reprogrammed into iPSCs, subcloned, and then transduced with the lentiviral RGB-BC system (Fig. 2a). Flow cytometry demonstrated that the composition of fluorescent labels remained relatively consistent over 17 passages (Additional file 4a-c). Sequencing of barcodes confirmed a rather stable composition of subclones in the iPSC lines of donors 1 and 2 (Fig. 2b, c). In contrast, iPSCs of donor 3 revealed a continuous decline in barcode complexity until only three barcodes constituted 61.8% of all barcodes at passage 17 (Fig. 2d). This is also reflected by a more pronounced decline of the Shannon Index in iPSCs of donor 3 (Fig. 2g). Overall, the composition of subpopulations remained relatively stable during the expansion of iPSCs.

### iMSCs are derived from few cells within iPSC preparations

We further analyzed how the clonal composition changes during the differentiation of iPSCs into iMSCs (Fig. 2a). Five weeks after transferring the iPSCs in MSC culture medium, the cells acquired a typical fibroblastoid and plastic adherent growth, an MSC-like immunophenotype (CD105^+^, CD73^+^, CD90^+^, CD29^+^, CD45^−^, CD34^−^, CD14^−^, CD31^−^), and in vitro differentiation potential towards adipogenic, osteogenic, and chondrogenic lineages. Therefore, all iMSCs were in line with the minimal criteria for the definition of MSCs [3] (Additional file 5). However, the iPSCs of donor 1 repeatedly stopped proliferating within the first 5 weeks of differentiation towards iMSCs. iMSCs of donors 2 and 3 could be further expanded until passage 12 and 8 after transduction, respectively, before entering growth arrest. Flow cytometry indicated that dominant iMSC clones arose after three to five passages after transduction (Additional file 4d-e). Genetic barcoding clearly validated clonal restriction in both iMSC preparations (Fig. 2e–f). Eight passages (56–66 days) after the induction of differentiation towards iMSCs about 85% and 72% of the iMSCs were derived from the three most prominent barcodes in donors 2 and 3, respectively. The Shannon Index revealed a clear drop in the clonal heterogeneity between passages 3 to 6 (Fig. 2g).

The selection of subpopulations might be due to chromosomal instabilities. Therefore, we analyzed copy number variations (CNV) with SNP arrays. This analysis revealed up to six chromosomal deletions or duplications, which were present in iPSCs and the corresponding iMSCs after long-term expansion (donor 2, P11; donor 3, P6; donor 1, P2 during the differentiation process due to proliferation abort). Depending on the formal thresholds applied to CNV analysis (chromosomal region size > 200 kb, mean marker distance < 5 kb), several variants were only detected in either iPSCs or corresponding iMSCs. However, in most cases, similar putative CNVs were observed in the corresponding cell type as well (Fig. 2h, Additional file 6). Only one 272 kb gain in chromosome 4q22.1 was detected in iPSCs but not in iMSCs of donor 3. To estimate the functional relevance of genes within the deletions and duplications (Additional file 7), we screened for cancer-associated genes in the Catalogue of Somatic Mutations in Cancer (COSMIC) Cancer Gene Census (CGC) [34]. The 20q11.21-gain in donor 1 comprised 96 genes, including the tumor suppressor gene *ASXL1* and the anti-apoptotic factor *BCL2L1.* The 12q13.12-loss (donor 2) contained *PRPF40B*, which has a moderate association with cancer, and the 4q28.1 gain (donor 3) comprised the tumor suppressor gene *FAT4*. Taken together, we did not detect any chromosomal variants that were acquired during differentiation towards iMSCs. Furthermore, none of the detected variants in iMSCs revealed apparent mosaicism in iPSCs. Thus, there was no evidence for driver mutations that result in the dominant clonal outgrowths of iMSCs—but the results clearly support the notion that genetic aberrations need to be taken into account for safety analysis of iPSC-derived cell preparations.

### Senescence-associated DNA methylation patterns in iMSCs

**Fig. 3:**
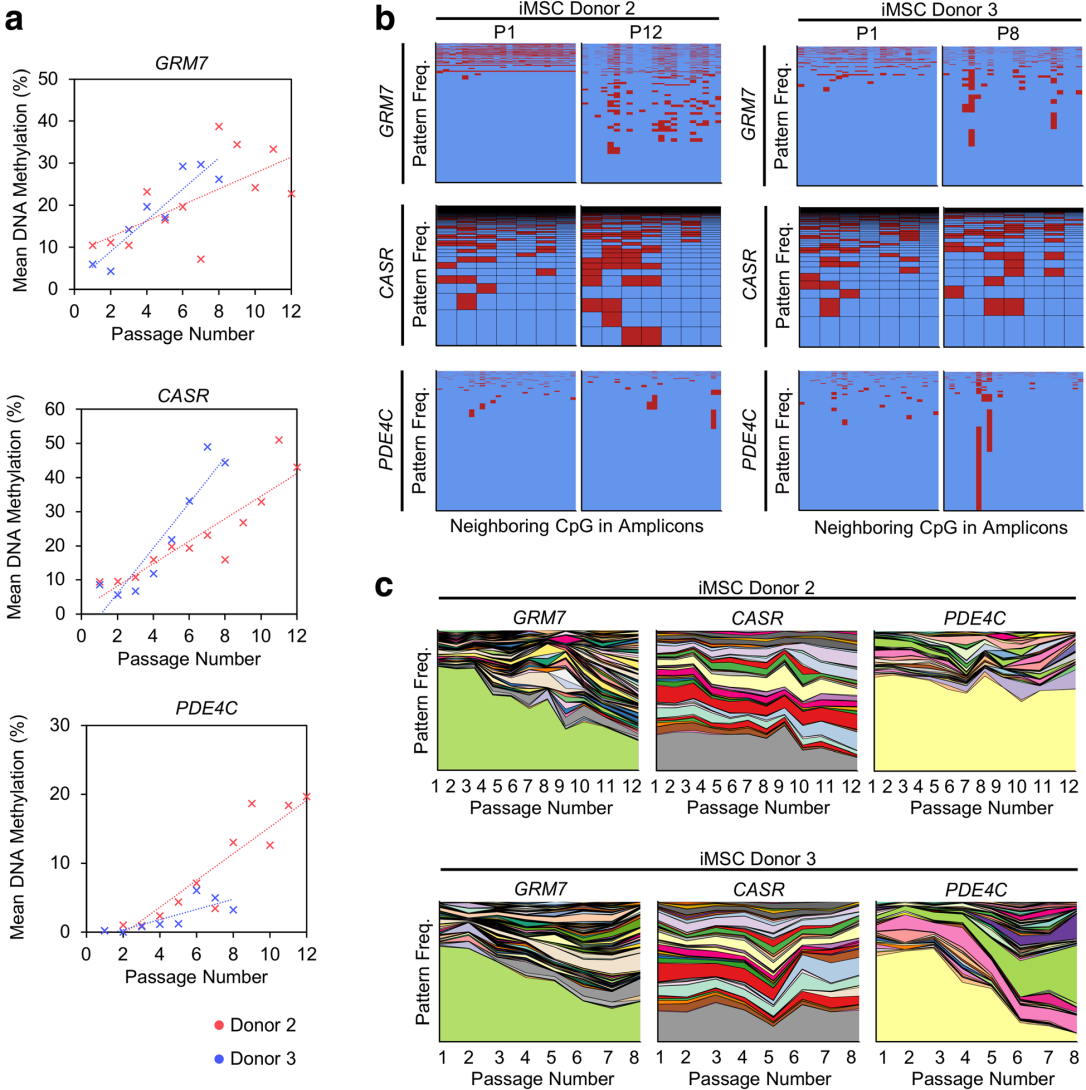
DNA methylation patterns do not clearly reflect subclones in iMSCs. **a** Bisulfite barcoded amplicon sequencing reveals changes in mean DNA methylation levels during expansion of iMSCs in *GRM7*, *CASR*, and *PDE4C* (representative CpG sites are depicted)*.***b** Frequencies of different DNA methylation patterns in individual reads of the amplicons of *GRM7*, *CASR*, and *PDE4C* of iMSCs at early versus late passages (red = methylated; blue = non-methylated). The height is indicative for the frequency of the corresponding pattern. P, passage. **c** Changes in the composition of different DNA methylation patterns during culture of iMSCs

Subsequently, we followed the hypothesis, that the prevalence of specific DNA methylation patterns, which are stochastically acquired during replicative senescence or aging, might also uncover the oligoclonal composition of subpopulations in iMSCs. We have exemplarily analyzed senescence-associated regions within the genes *GRM7* (22 neighboring CpG sites) and *CASR* (7 neighboring CpG sites) by barcoded bisulfite amplicon sequencing (BBA-seq). Overall, CpGs within these two regions revealed continuous changes in DNA methylation during culture expansion (Fig. 3a). The analysis of DNA methylation patterns within individual BBA-seq reads of *GRM7* and *CASR* hardly revealed any patterns that became dominant during the expansion of iMSCs (Fig. 3b, c). Alternatively, we analyzed an age-associated genomic region within the gene *PDE4C* (26 neighboring CpG sites)*,* which was recently demonstrated to reflect clonal growth in myeloid malignancies [18]*.* In fact, one specific DNA methylation pattern within *PDE4C* became dominant in iMSCs of donor 3. In MSCs or iPSCs, the comparison of early versus late passages did not reveal dominant DNA methylation patterns (Additional file 8). Taken together, the analysis of dominant DNA methylation patterns did not provide a reliable alternative to genetic barcoding to detect dominant subclones.

### Limiting dilution analysis of MSC and iMSC populations

**Fig. 4:**
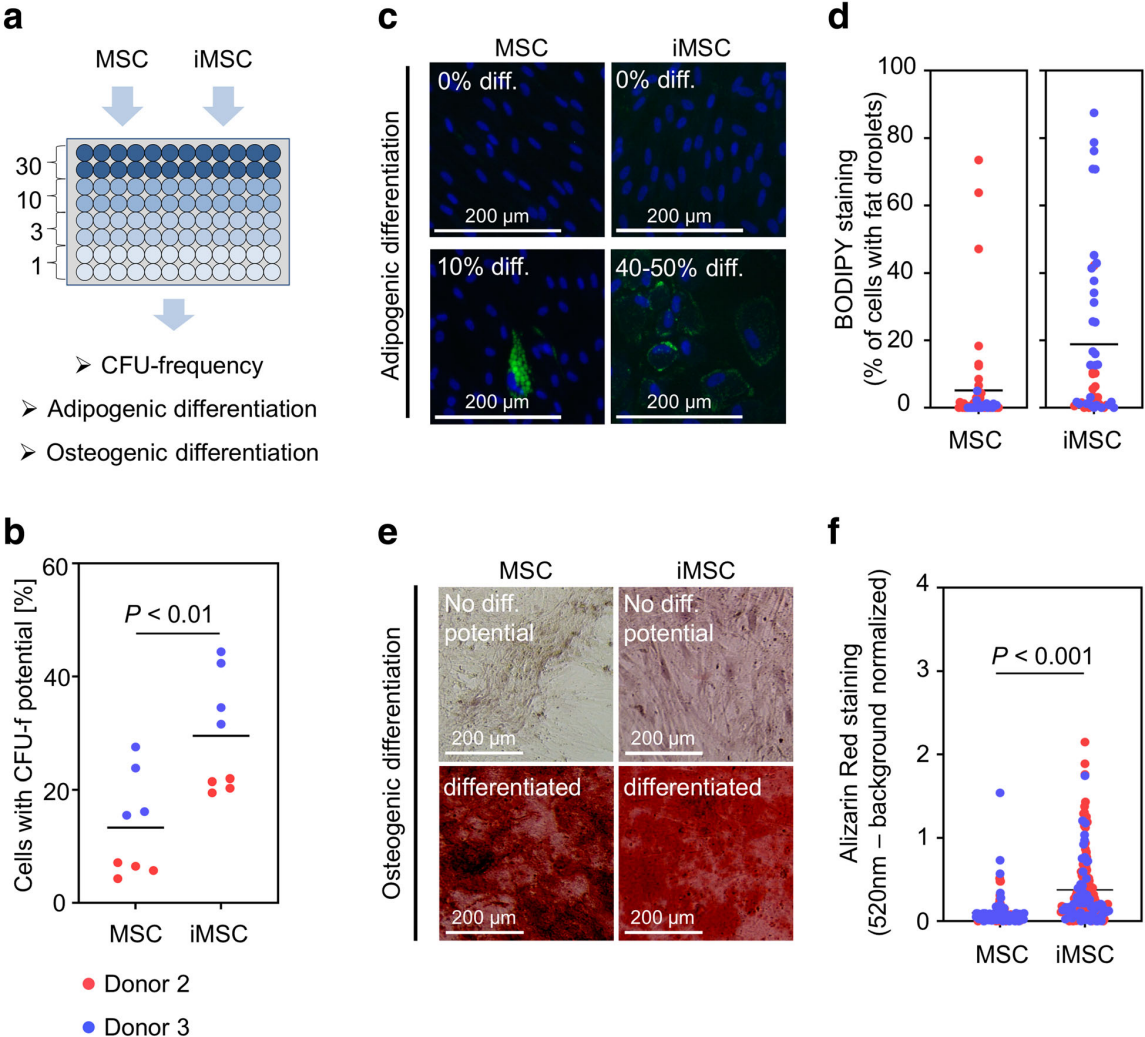
Functional analysis of subpopulations in MSCs and iMSCs. **a** Schematic presentation of limiting dilution assays. Either primary MSCs or iMSCs of two corresponding donors were used. Cells were seeded on four 96-well plates at decreasing concentrations (30, 10, 3, and 1 cells/well). After 15 days of incubation, wells were scored for at least 50% confluency to determine CFU-f frequencies. The same plates were then induced towards adipogenic and osteogenic lineage to compare in vitro differentiation potential. **b** Analysis of CFU-f frequencies of MSCs and iMSCs (*N* = 2; *n* = 4). **c** Representative pictures of subclones without and with adipogenic differentiation potential (BODIPY/Hoechst staining 14 days after induction of differentiation; donor 3). **d** For individual MSC and iMSC subclones, the percentage of cells with fat droplet formation upon adipogenic differentiation was analyzed. Due to different morphology of fat droplets the results were not directly compared in one diagram. **e** Representative pictures of subclones without and with osteogenic differentiation potential (Alizarin Red staining 18 days after induction of osteogenic differentiation; donor 3). **f** Osteogenic differentiation was quantified for individual subclones in MSCs and iMSCs. Alizarin Red staining was measured by background normalized absorption

Next, we compared growth and differentiation potential in subclones of MSCs and iMSCs by seeding the cells in limiting dilutions in multi-well plates (Fig. 4a). Since iMSCs were observed as early as 4 to 5 weeks after changing the culture conditions from iPSC to MSC medium, we hypothesized that iMSCs after 5 weeks would roughly correspond to primary MSCs of passage 2 [9, 35]. As mentioned before, iPSCs of donor 1 repeatedly failed to differentiate towards iMSCs, and therefore, this donor was not considered for this analysis. After 15 days, we scored the wells for colony formation to estimate CFU-f potential by Poisson statistics: for primary MSCs, the CFU-f frequency was 5.8% ± 1.2% and 20.7% ± 5.9% for donors 2 and 3, respectively. In contrast, iMSCs from the same donors had a significantly higher colony-forming potential (20% ± 1.1% for donor 2 and 38.2% ± 6.1% for donor 3; *P* = 0.01; Fig. 4b).

Subsequently, we differentiated the multi-well plates with colonies either towards adipogenic or osteogenic lineages for an additional 2 weeks. For this analysis, we only considered wells at a dilution that statistically resembled single-cell-derived clones. In primary MSCs, only 17% of clones in donor 2 and none in donor 3 revealed a clear and rather homogeneous adipogenic differentiation (> 10% of cells with fat droplets; Fig. 4c, d), which is in line with a previous study [1]. In iMSCs, the acquisition of fat droplets was less prominent, as described before [9–11, 35]. However, counting all cells with detectable fat droplets as positive, the average percentage of droplet-forming cells was higher in iMSC clones than in MSC clones. When we analyzed the percentage of single-cell-derived iMSC clones that revealed clear adipogenic differentiation (> 10% of cells with fat droplets), this was observed in 25% of iMSC clones in donor 2 and 62% of iMSC clones in donor 3. In analogy, we analyzed osteogenic differentiation by Alizarin Red staining of calcium phosphate precipitates. Primary MSCs as well as iMSCs comprised subclones with and without osteogenic differentiation potential (Fig. 4e). In primary MSCs, only about 4.6% of all subclones clearly demonstrated calcium phosphate precipitates, while in iMSCs, this percentage was higher with 30.9% (Fig. 4f). Thus, MSCs and iMSCs both comprised heterogeneous subclones, but the CFU-f frequency and the fraction of multipotent cells seemed to be higher in iMSC preparations.

## Discussion

Culture expansion of cells in vitro may favor clonal dominance, which potentially entails capturing a specific mutational and epigenetic background. Therefore, the clonal composition needs to be taken into account for cellular therapy. For various different cell types and cell lines, it has been demonstrated that there is generally a continuous, discreet loss in the subclonal diversity due to cell splitting and subtle genetic and epigenetic differences [36]. Cai and coworkers followed clonal dynamics at consecutive passages of MSCs by tracking of genetic variances with whole genome sequencing and droplet digital PCR [37]. Their results indicated that MSCs maintain a stable genomic composition in the early passages but are subject to clonal growth upon extended expansion. Furthermore, Selich et al. demonstrated a strong selection of MSC subpopulations during the initial isolation from umbilical cord pieces [7]. In contrast, our results reveal only a slow, gradual loss of subsets in bone marrow-derived MSCs. The reason why we did not observe massive clonal selection might be due to the fact that we did not label the cells in tissue but used already established MSC preparations. Furthermore, we could only track cells for a limited number of passages (until P6-P8) before they entered a senescent state, which might be due to the lentiviral transduction and puromycin selection.

For iPSCs, we observed an even lower clonal restriction over up to 17 passages—albeit the Shannon Index also declined continuously. Brenière-Letuffe et al. [38] have recently followed the clonal dynamics in iPSCs upon RGB-labeling by fluorescence analysis at regular intervals. Their results indicated that after about 15 passages iPSCs reached oligoclonality, without overt evidence for genetic changes. Since every cell passage corresponds to a random selection of cells, the impact on clonal dynamics might be reduced by larger cell culture flasks and usage of cell stocks of early passages [38].

While the decline of clonal diversity was relatively continuous in MSCs and iPSCs, we observed a sharp drop in the Shannon Index after 3 to 5 weeks of differentiation towards iMSCs. The clonal dominance within iMSCs of donor 3 might also be reflected by prevalent DNA methylation patterns in *PDE4C*. However, this method did not correlate well with RGB-BC results and, thus, does not appear reliable to track the clonal dynamics of cells in culture. Furthermore, our mutational analysis did not identify genetic aberrations that were acquired during the differentiation of iPSCs towards iMSCs. Thus, the clonal restriction that we observed in iMSCs of donors 2 and 3 does not appear to be due to an additional mutation with growth advantage. It rather seems to be caused by an insufficient and non-directed differentiation procedure that favors the dominance of few individual iMSC clones.

Genetic integrity is crucial for cellular therapeutics. Many other studies reported a high number of genetic abnormalities in human pluripotent stem cell (hPSC) lines, which often arise recurrently [39]. In fact, all three iPSC clones used in this study revealed chromosomal duplications and deletions, which were passed on to the corresponding iMSCs. The 20q11.21 gain that we found in donor 1 has been identified in 20% of all hPSC lines [40]. This region includes the gene *BCL2L1*, an anti-apoptotic factor that favors clonal outgrowth [40, 41]. Notably, this genetic aberration may also impair the differentiation of pluripotent cells [41], and this might explain why iPSCs of donor 1 repeatedly failed to differentiate towards iMSCs.

Despite their homogeneous appearance, MSCs are composed of subsets with different growth and differentiation potential, and our results demonstrate that the same also applies for iMSCs. At passage 2, our bone marrow-derived MSCs revealed a CFU-f frequency of about 0–30%, which is in the range of previous studies [1, 4, 5, 37]. For iMSCs at a corresponding passage, we observed slightly higher CFU-f frequencies, which have also been suggested by Zhao et al. [35]. Comparison of individual colonies indicated that more subsets are capable to differentiate towards osteogenic and adipogenic lineage in iMSCs versus MSCs. Either way, iMSCs clearly revealed heterogeneous proliferation and in vitro differentiation potential.

A better understanding of how the clonal composition of cell preparations changes during culture expansion is crucial for clinical therapy [42]—particularly in the light of ongoing studies with iMSCs for steroid-refractory graft versus host disease [12, 13]. To date, it remains a challenge to establish a framework of safety standards for hPSC-derived cells [43]. Unlike iPSCs, tumorigenesis is usually not induced by iMSCs in the teratoma formation assay [44]. However, during culture expansion, iMSCs undergo replicative senescence-like primary MSCs. Accordingly, we have recently demonstrated that MSCs and iMSCs entered a senescent state after 21.3 ± 1.4 and 17.1 ± 3.8 cumulative population doublings, respectively [45]. Furthermore, this process is associated with very similar changes in morphology, differentiation potential, expression of senescence-associated beta-galactosidase, transcriptome, epigenome, and metabolome [45]. It is conceivable that this process of replicative senescence contributes to the dynamics within subpopulations during culture expansion.

Despite the great potential of genetic barcoding to understand these processes, it needs to be taken into account that not every cell is labeled and that additional selection with puromycin may further foster the process of cellular aging. While this method cannot be directly applied to cellular therapeutic products due to potential insertion mutagenesis, it will shed further light into how the clonal composition is affected by culture and differentiation regimen.

## Conclusions

Taken together, our genetic barcoding approach demonstrates that the subclonal diversity of MSCs and iPSCs only gradually declines during expansion. In contrast, the derivation of iMSCs from iPSCs may be based on a few individual cells, and this entails higher proliferative pressure and may favor the capturing of genetic aberrations. Thus, there is a need to develop a more effective differentiation regimen that better supports homogeneous and polyclonal differentiation towards iMSCs.

## Supplementary information

**Additional file 1.** Barcode-oligonucleotides. Random and color specific nucleotides are marked in red. (XLS 20 kb)

**Additional file 2. **RGB-BC lentiviral vector construct and flow cytometry of MSCs. **(a)** The RGB-BC lentiviral vectors contain one of three fluorophores (mCherry, red; Venus, yellow-green; or Cerulean, blue), which are driven by the Cbx3/EFS promoter. The insert regions additionally contain a barcode (B) comprising 15 color-specific and 16 random (N) nucleotides. The plasmids were modified from Selich et al. (2019) by integrating a puromycin resistance transgene (Puro) with a 2A self-cleaving peptide (P2A) for the selection of transduced cells. LTR = long terminal repeat, Δ = self-inactivating U3 deletion, R = repeat region, U5 = unique 5' region, wPRE = woodchuck hepatitis virus posttranscriptional regulatory element, Cbx3/EFS = chromobox protein homolog 3/elongation factor 1α short. **(b-d)** The expression of the fluorophores of the RGB-BC lentiviral vectors was analyzed during culture expansion of three MSC preparations. The composition of fluorochromes was estimated as indicated in the legend. The fraction of non-fluorescent clones might be overestimated due to thresholds to reduce activation by other fluorochromes. Overall, the frequencies of fluorophore-combinations remained relatively consistent during expansion of MSCs.

**Additional file 3.** Primers. Handle sequences of the primers for the barcoding PCR of the MiSeq library preparation are marked in red. (XLS 28 kb)

**Additional file 4. **Flow cytometry of the clonal diversity in iPSCs and iMSCs. **(a-c)** The expression of the fluorophores of the RGB-BC lentiviral vectors was analyzed during culture expansion of three iPSC preparations. Overall, the frequencies of fluorophore-combinations remained relatively constant throughout 17 passages. **(d-e)** Flow cytometry of cellular subsets in iMSCs. At passage 1 the RGB-BC labelled iPSCs (as in A-C) were induced towards iMSCs. However, the iPSCs of donor 1 reproducibly stopped proliferation within four to five passages during the differentiation procedure and were therefore not depicted here. The iMSCs of donors 2 and 3 revealed dominant subsets after four to five passages, which became non-fluorescent, possibly due to gene silencing. * = Samples with less than 3,000 events in the forward- and side-scatter gates for fluorescence analysis.

**Additional file 5. **Characterization of iMSCs. **(a)** Exemplary morphological changes of iPSCs of donor 2 during the differentiation process towards iMSCs. Within four to five weeks, the cells acquired typical fibroblastoid morphology. **(b)** Primary MSCs (for control; passage 2) and iMSCs (passage 6) were induced towards adipogenic, osteogenic and chondrogenic lineages, and then stained with BODIPY/Hoechst, Alizarin Red, or Alcian blue/PAS, respectively. Exemplary images are presented for donor 2. **(c)** Immunophenotypic comparison of surface marker expression in MSCs and iMSCs. The histograms depict exemplary flowcytometric measurements of donor 2. The biphasic peak in CD90 expression of iMSCs was repeatedly observed, while the peak with lower expression declined during culture expansion (not depicted). Overall, iMSCs fulfilled the minimal criteria for the definition of MSCs.

**Additional file 6.** Copy number variations in iPSCs and iMSCs as detected by SNP arrays. Aberrations as detected by SNP arrays with thresholds of size >200kbp and mean marker distance <5kbp are depicted in black. Corresponding chromosomal regions in the other cell type below these threshold values are marked in red. In almost all cases, corresponding mutations were present, but often below threshold values. (XLS 22 kb)

**Additional file 7.** Genes of genomic regions with deletions and duplications in iPSCs and iMSCs. This table provides gene IDs for each genetic aberration observed in CNV analysis. (XLS 36 kb)

**Additional file 8. **DNA methylation patterns in iPSC and MSC populations during culture expansion. **(a)** Frequencies of different DNA methylation patterns in individual reads of the amplicons of GRM7, CASR, and PDE4C, in iPSCs of donors 2 and 3 in early versus late passages. (red = methylated; blue = non-methylated). The height is indicative for the frequency of the corresponding pattern. **(b)** Frequencies of different DNA methylation patterns in early versus late passages of MSCs of donors 2 and 3.

## Data Availability

The datasets generated and analyzed during the current study are available from the corresponding author on reasonable request.

## Abbreviations

MSCs: Mesenchymal stromal cells
iPSCs: Induced pluripotent stem cells
iMSCs: iPSC-derived MSCs
CFU-f: Fibroblastic colony forming units
RGB: Red-green-blue
RGB-BC: Multicolor lentiviral genetic barcode labeling
UMIs: Unique molecular identifiers
CpGs: CG dinucleotides
DMEM: Dulbecco’s modified Eagle’s medium
EDTA: Ethylenediaminetetraacetic acid
BODIPY: 4,4-Difluoro-1,2,5,7,8-pentamethyl-4-bora-3a,4a-diaza-s-indacene
FACS: Fluorescence-activated cell sorter
PE: Phycoerythrin
APC: Allophycocyanin
FITC: Fluorescein isothiocyanate
LeGO: Lentiviral Gene Ontology
VSV-G: Vesicular stomatitis virus G glycoprotein
HEK293T: Human embryonic kidney 293 T cell line
PFA: Paraformaldehyde
PBS: Phosphate-buffered saline
SNP: Single nucleotide polymorphism
P: Passage
CNV: Copy number variation
GRM7: Glutamate metabotropic receptor 7 gene
CASR: Calcium-sensing receptor gene
PDE4C: Phosphodiesterase 4C gene
BBA-seq: Barcoded bisulfite amplicon sequencing
