# Supplementary figures and images for: Genetic barcoding reveals clonal dominance in iPSC-derived mesenchymal stromal cells
Source: Stem Cell Res Ther. 2020 Mar 5;11:105. doi: 10.1186/s13287-020-01619-5 (PMC7059393; doi:10.1186/s13287-020-01619-5)

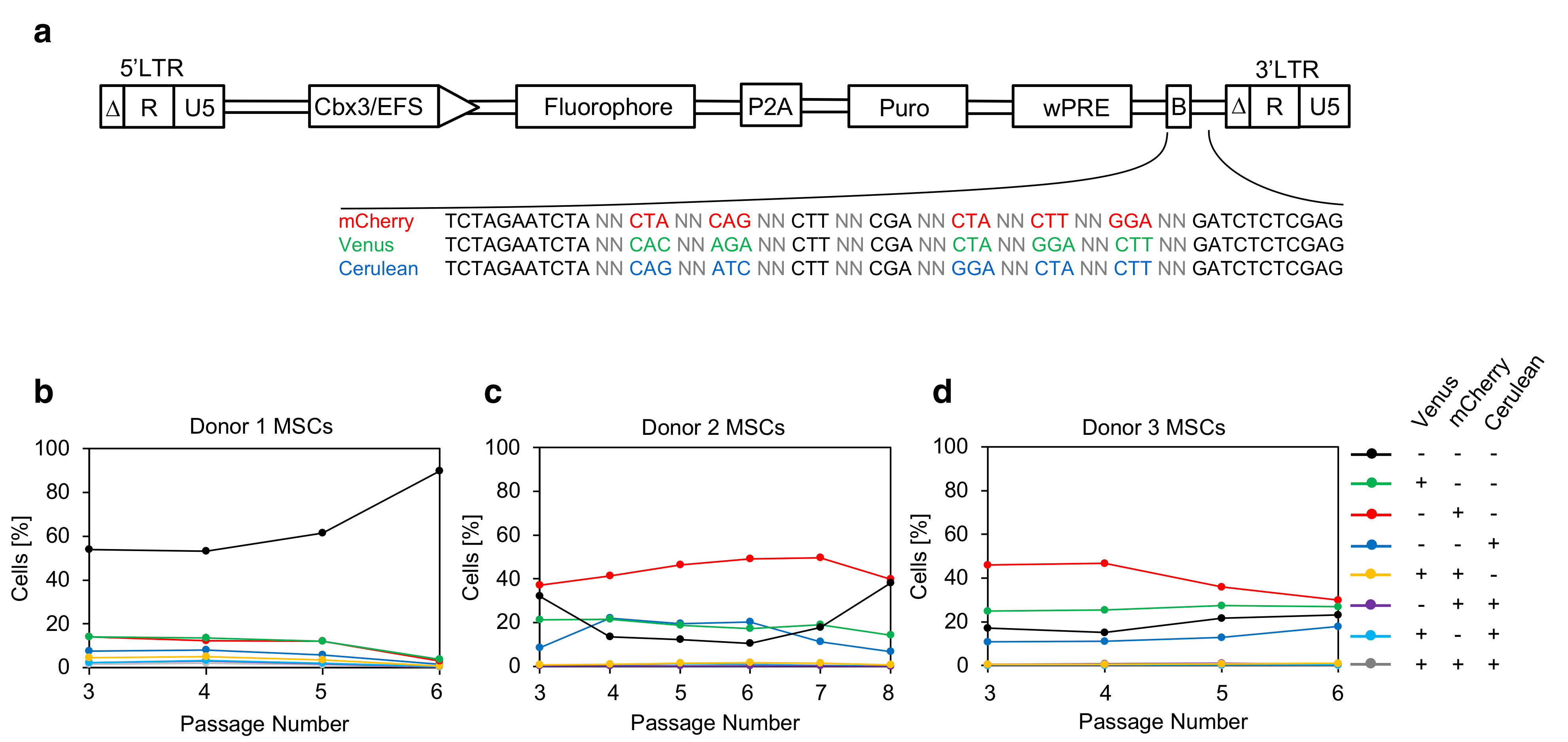

Supplement: Supplementary file 2 — Additional file 2. RGB-BC lentiviral vector construct and flow cytometry of MSCs. (a) The RGB-BC lentiviral vectors contain one of three fluorophores (mCherry, red; Venus, yellow-green; or Cerulean, blue), which are driven by the Cbx3/EFS promoter. The insert regions additionally contain a barcode (B) comprising 15 color-specific and 16 random (N) nucleotides. The plasmids were modified from Selich et al. (2019) by integrating a puromycin resistance transgene (Puro) with a 2A self-cleaving peptide (P2A) for the selection of transduced cells. LTR = long terminal repeat, Δ = self-inactivating U3 deletion, R = repeat region, U5 = unique 5' region, wPRE = woodchuck hepatitis virus posttranscriptional regulatory element, Cbx3/EFS = chromobox protein homolog 3/elongation factor 1α short. (b-d) The expression of the fluorophores of the RGB-BC lentiviral vectors was analyzed during culture expansion of three MSC preparations. The composition of fluorochromes was estimated as indicated in the legend. The fraction of non-fluorescent clones might be overestimated due to thresholds to reduce activation by other fluorochromes. Overall, the frequencies of fluorophore-combinations remained relatively consistent during expansion of MSCs. [file 13287_2020_1619_MOESM2_ESM.tif]

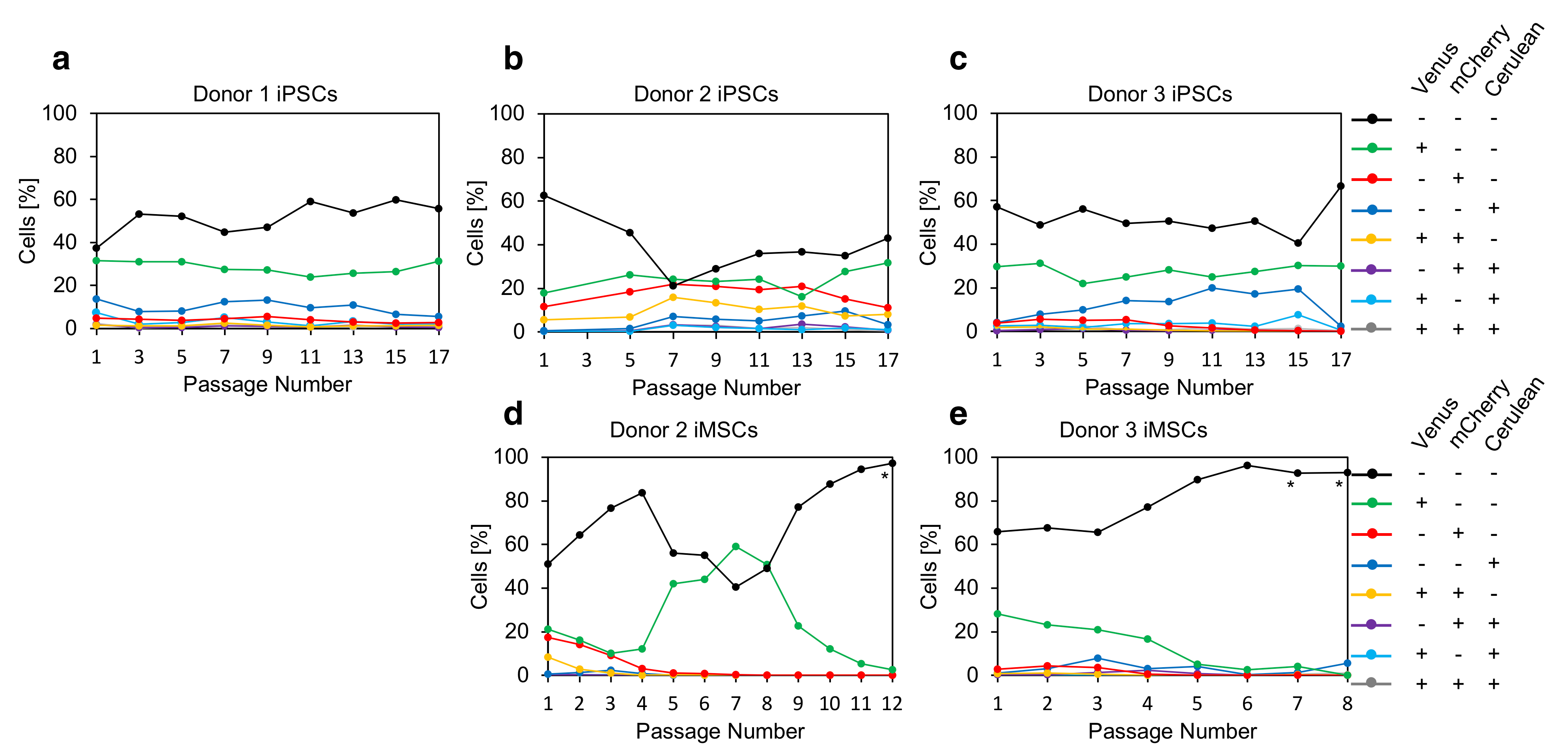

Supplement: Supplementary file 4 — Additional file 4. Flow cytometry of the clonal diversity in iPSCs and iMSCs. (a-c) The expression of the fluorophores of the RGB-BC lentiviral vectors was analyzed during culture expansion of three iPSC preparations. Overall, the frequencies of fluorophore-combinations remained relatively constant throughout 17 passages. (d-e) Flow cytometry of cellular subsets in iMSCs. At passage 1 the RGB-BC labelled iPSCs (as in A-C) were induced towards iMSCs. However, the iPSCs of donor 1 reproducibly stopped proliferation within four to five passages during the differentiation procedure and were therefore not depicted here. The iMSCs of donors 2 and 3 revealed dominant subsets after four to five passages, which became non-fluorescent, possibly due to gene silencing. * = Samples with less than 3,000 events in the forward- and side-scatter gates for fluorescence analysis. [file 13287_2020_1619_MOESM4_ESM.tif]

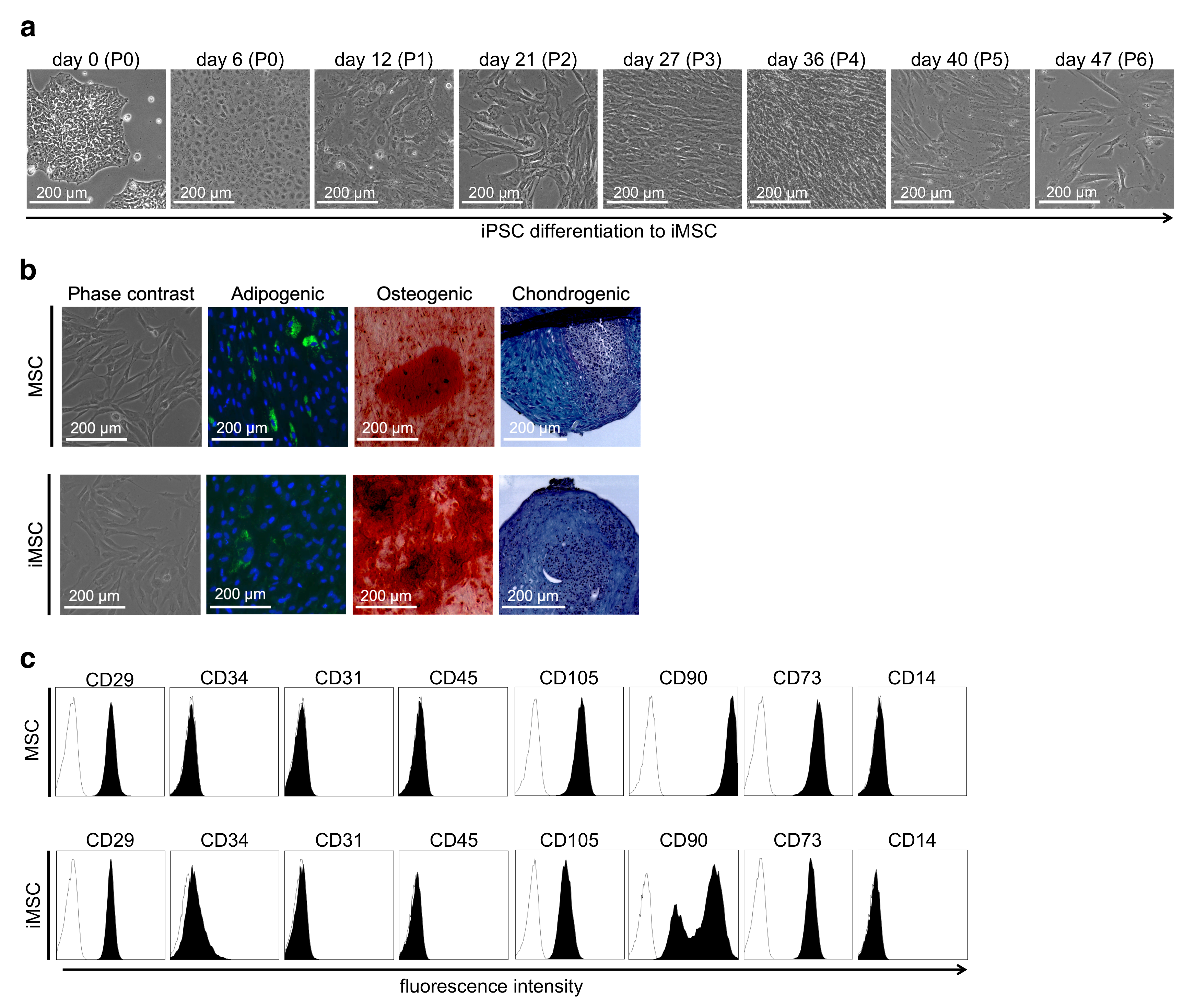

Supplement: Supplementary file 5 — Additional file 5. Characterization of iMSCs. (a) Exemplary morphological changes of iPSCs of donor 2 during the differentiation process towards iMSCs. Within four to five weeks, the cells acquired typical fibroblastoid morphology. (b) Primary MSCs (for control; passage 2) and iMSCs (passage 6) were induced towards adipogenic, osteogenic and chondrogenic lineages, and then stained with BODIPY/Hoechst, Alizarin Red, or Alcian blue/PAS, respectively. Exemplary images are presented for donor 2. (c) Immunophenotypic comparison of surface marker expression in MSCs and iMSCs. The histograms depict exemplary flowcytometric measurements of donor 2. The biphasic peak in CD90 expression of iMSCs was repeatedly observed, while the peak with lower expression declined during culture expansion (not depicted). Overall, iMSCs fulfilled the minimal criteria for the definition of MSCs. [file 13287_2020_1619_MOESM5_ESM.tif]

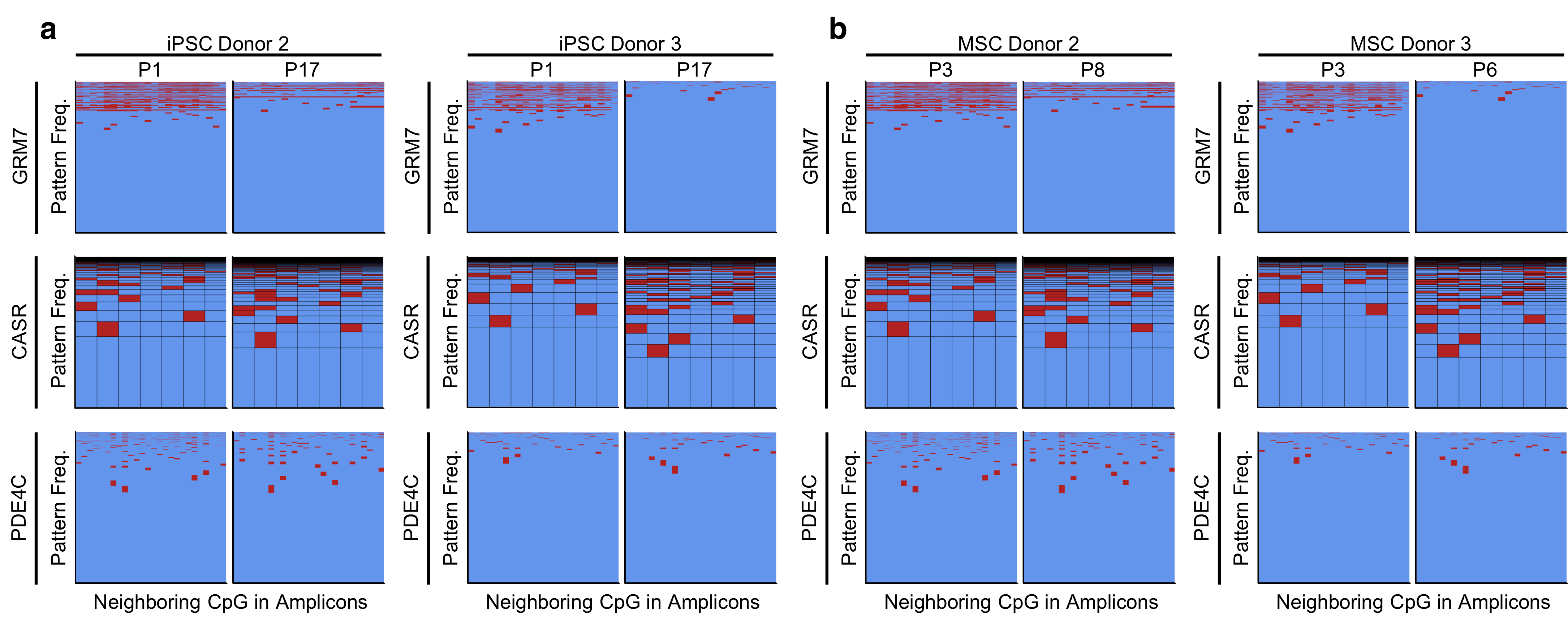

Supplement: Supplementary file 8 — Additional file 8. DNA methylation patterns in iPSC and MSC populations during culture expansion. (a) Frequencies of different DNA methylation patterns in individual reads of the amplicons of GRM7, CASR, and PDE4C, in iPSCs of donors 2 and 3 in early versus late passages. (red = methylated; blue = non-methylated). The height is indicative for the frequency of the corresponding pattern. (b) Frequencies of different DNA methylation patterns in early versus late passages of MSCs of donors 2 and 3. [file 13287_2020_1619_MOESM8_ESM.tif]
